# Combined inhibition of DNA methylation and histone acetylation enhances gene re-expression and drug sensitivity *in vivo*

**DOI:** 10.1038/sj.bjc.6604932

**Published:** 2009-03-03

**Authors:** N Steele, P Finn, R Brown, J A Plumb

**Affiliations:** 1 Centre for Oncology and Applied Pharmacology, University of Glasgow, CRUK Beatson Laboratories, Garscube Estate, Glasgow G61 1BD, UK; 2 TopoTarget Prolifix Ltd, Abingdon, Oxford OX14 4RY, UK

**Keywords:** DNA methylation, MLH1, drug sensitivity, decitabine, histone deacetylase inhibitor

## Abstract

Histone deacetylation and DNA methylation have a central role in the control of gene expression in tumours, including transcriptional repression of tumour suppressor genes and genes involved in sensitivity to chemotherapy. Treatment of cisplatin-resistant cell lines with an inhibitor of DNA methyltransferases, 2-deoxy-5′azacytidine (decitabine), results in partial reversal of DNA methylation, re-expression of epigenetically silenced genes including *hMLH1* and sensitisation to cisplatin both *in vitro* and *in vivo*. We have investigated whether the combination of decitabine and a clinically relevant inhibitor of histone deacetylase activity (belinostat, PXD101) can further increase the re-expression of genes epigenetically silenced by DNA methylation and enhance chemo-sensitisation *in vivo* at well-tolerated doses. The cisplatin-resistant human ovarian cell line A2780/cp70 has the *hMLH1* gene methylated and is resistant to cisplatin both *in vitro* and when grown as a xenograft in mice. Treatment of A2780/cp70 with decitabine and belinostat results in a marked increase in expression of epigenetically silenced MLH1 and MAGE-A1 both *in vitro* and *in vivo* when compared with decitabine alone. The combination greatly enhanced the effects of decitabine alone on the cisplatin sensitivity of xenografts. As the dose of decitabine that can be given to patients and hence the maximum pharmacodynamic effect as a demethylating agent is limited by toxicity and eventual re-methylation of genes, we suggest that the combination of decitabine and belinostat could have a role in the efficacy of chemotherapy in tumours that have acquired drug resistance due to DNA methylation and gene silencing.

Epigenetic inactivation of genes crucial for control of normal cell growth is a hallmark of cancer cells (Hanahan and Weinberg, 2000). These epigenetic mechanisms include crosstalk between DNA methylation, histone modification and other components of chromatin higher order structure leading to regulation of gene transcription. The transfer of a methyl group to the carbon 5 position of cytosines, almost always in the context of CpG dinucleotides, is the only known epigenetic modification of DNA itself in mammalian cells. Many tumours show increased methylation of CpG islands, CpG rich regions of DNA usually although not exclusively associated with gene promoters, which is associated with epigenetic silencing (Brown and Strathdee, 2002). CpG islands aberrantly methylated in tumours are associated with silencing of genes involved in control of the cell cycle, apoptosis and drug sensitivity as well as tumour suppressor genes (Brown and Plumb, 2004).

Epigenetic silencing occurring during tumour development has the potential to affect the chemo-sensitivity of tumour cells and how tumours will respond to chemotherapy (Teodoridis *et al*, 2005). One example of this is epigenetic inactivation of the CpG island at the DNA repair gene, MGMT and response of glioma to monofunctional alkylating agents such as temozolomide (Hegi *et al*, 2005). DNA methylation and epigenetic silencing of tumour cells can also be selected for during chemotherapy and may be an important driving force behind acquired drug resistance (Glasspool *et al*, 2006). The MLH1 protein, part of the human DNA mismatch repair system, has been shown to be important in determining sensitivity to a number of important chemotherapeutic agents including alkylating agents and cisplatin (Fink *et al*, 1998; Brown, 1999). Loss of mismatch repair due to methylation of the *hMLH1* gene promoter results in resistance to cisplatin in cell lines *in vitro* and in human tumour xenografts *in vivo* (Plumb *et al*, 2000). Methylation of the *hMLH1* gene promoter is observed in many tumour types (Strathdee *et al*, 1999; Gifford *et al*, 2004) and loss of MLH1 expression is associated with clinical drug resistance in ovarian cancer (Samimi *et al*, 2000).

There is considerable interest in the potential to use epigenetic therapies in combination with existing chemotherapeutic agents, both for improving initial tumour response and for overcoming acquired drug resistance. We have previously shown that treatment of ovarian and colon cell lines with the DNA hypomethylating agent 2-deoxy-5′azacytidine (decitabine, DAC) results in partial reversal of DNA methylation, re-expression of methylated loci such as *hMLH1* and sensitisation to cisplatin and carboplatin both *in vitro* and *in vivo* (Plumb *et al*, 2000). In studies of human tumour xenografts in mice we were able to demonstrate that decitabine treatment reduced the level of methylation of the *hMLH1* gene promoter and that this was associated with re-expression of MLH1 in a small proportion of the tumour cells at doses that clearly conferred increased sensitisation and were well tolerated. In a phase 1 clinical trial of decitabine in combination with carboplatin in advanced solid tumours, a reduction in methylcytosine content of PBMCs was observed that was comparable to that observed in mice where chemo-sensitisation of xenografts occurred (Appleton *et al*, 2007). However, limited demethylation of tumour was observed. The dose limiting toxicity of decitabine was identified as myelosuppression and this toxicity plus the limited demethylation in tumours and eventual re-methylation of genes may limit the clinical use of decitabine when used alone in solid tumours.

Baylin and co-workers have shown that the combination of the histone deacetylase (HDAC) inhibitor trichostatin A with decitabine is more effective in reactivating transcription of epigenetically silenced genes such as *hMLH1* in tumour cell lines than either drug alone (Cameron *et al*, 1999). The combination of a demethylating agent and an HDAC inhibitor has now been examined in clinical trials of haematological malignancies. However, in solid tumours it may be that epigenetic therapies may be more effective when used in combination with cytotoxic agents. We have therefore investigated whether it is possible to use a low, non-toxic dose of decitabine in combination with an inhibitor of histone deacetylase activity, belinostat (PXD101), to enhance the level of re-expression of epigenetically silenced genes in drug-resistant A2780/cp70 both *in vitro* and *in vivo* and whether this combination enhances chemo-sensitisation of xenografts.

## Materials and methods

### Cell lines

Cell line A2780/cp70 is an *in vitro* derived cisplatin-resistant variant of the ovarian cancer cell line A2780 originally obtained from Dr RF Ozols (Fox Chase Cancer Centre, Philadelphia, PA, USA). Cells were grown in RPMI-1640 supplemented with glutamine (2 mM) and FCS (10%). A2780/cp70 is mismatch repair deficient and does not express MLH1 due to hypermethylation of the *hMLH1* gene promoter (Strathdee *et al*, 1999) as well as having a number of other loci hypermethylated (Leu *et al*, 2003).

### Protein expression

For western blotting cells were grown in 25 cm^2^ flasks and exposed to drugs as specified. Cells were harvested with trypsin/EDTA and washed two times with ice cold PBS. They were resuspended in 200 *μ*l of lysis buffer (50 mM Hepes pH 7.0, 250 mM NaCl, 0.5% NP40) supplemented with protease inhibitors (Complete from Roche Diagnostics Ltd, Lewes, UK) and incubated on ice for 20 min. Samples were centrifuged at 12 000 **g** for 5 min at 4°C to remove debris. Supernatant proteins (20 *μ*g) were separated by the NuPage electrophoresis system (Invitrogen, Paisley, UK) on 4–12% Bis-Tris gels with 4-morpholinepropanesulfonic acid SDS running buffer. The ‘Novex Xcell II’ blotting apparatus (Invitrogen) was used to transfer proteins onto Immobilon polyvinylidene difluoride membrane (Millipore, Watford, UK). The membrane was blocked for 1 h in Tris-buffered saline containing 0.02% Tween 20 and 5% powdered milk and then incubated overnight at 4°C with the primary antibody (MLH1 from Pharmingen, BD UK Ltd, Oxford, UK, MAGE-A1 from Neomarkers, Lab Vision Ltd, Cambridge, UK). The membrane was then washed and incubated for 1 h at room temperature with the secondary antibody (sheep anti-mouse HRP, Amersham, GE Healthcare, Amersham, UK). After washing, the membrane protein bands were visualised by enhanced chemiluminescence (ECL, Amersham). Band intensity was quantified by densitometry (GS-800, Bio-Rad, Hemel Hempstead, UK).

For immunohistochemistry, tumours were fixed in neutral-buffered formalin and processed as previously described (Plumb *et al*, 2000).

### Human tumour xenografts

Animal studies were carried out under an appropriate United Kingdom Home Office Project Licence and all work conformed to the UKCCR Guidelines for the welfare of animals in experimental neoplasia. Monolayer cultures were harvested with trypsin/EDTA and resuspended in PBS. About 10^7^ cells were injected subcutaneously into the right flank of athymic nude mice (CD1 *nu/nu* mice from Charles River, Margate, UK). After 7–10 days when the mean tumour diameter was at ⩾0.5 cm, animals were randomised in groups of six for experiments. Standard sterile clinical formulations of cisplatin (Western Infirmary Pharmacy, Glasgow) decitabine (Supergen, Dublin, California, CA, UK) and belinostat (TopoTarget, Abingdon, UK) were used. Where specified, mice were pretreated with decitabine 6 days before cisplatin (6 mg kg^−1^ intraperitoneally), when tumours were just visible. Decitabine (5 mg kg^−1^) was administered intraperitoneally at 10:00, 13:00 and 16:00 hours (total dose 15 mg kg^−1^ per mouse). Belinostat (40 mg kg^−1^) was administered intraperitoneally 3 days before cisplatin where specified. Mice were weighed daily and tumour volumes were estimated by caliper measurements assuming spherical geometry (volume=*d*^3^ × π/6).

### Pyrosequencing

The methylation status of specific cytosine residues in the MAGE-A1 gene promoter was determined following bisulphite modification of DNA extracted from tumours. Tumours were removed from mice and snap frozen in liquid nitrogen. Frozen tumours were powdered in a ‘Mikro-Dismembrator’ homogeniser and DNA extracted with a BACC2 Nucleon extraction kit (Nucleon). Bisulphite modification was carried out with the CpGenome DNA modification kit (Chemicon International, Millipore, Watford, UK) according to the manufacturer's instructions. The modified DNA was amplified by PCR with primers chosen to bracket the CpG island of the MAGE-A1 gene promoter (forward PCR: 5′-TTTTTATTTTTATTTAGGTAGGAT-3′ and reverse PCR: Biotin-5′-TCTAAAAACAACCCAAACTAAAAC-3′). The PCR was carried out in 50 *μ*l volumes containing 2 U Faststart Taq polymerase, 10 × Faststart Buffer (Roche), 10 mM dNTPs (Applied Biosystems, Warrington, UK), 3.5 mM MgCl_2_ (Roche), oligonucleotides (Biomers, www.biomers.net) at 1 *μ*M and 2 *μ*l of modified DNA template. A 40-*μ*l PCR product was used for pyrosequencing according to the manufacturer's instruction. Sixteen picomoles of the sequencing primers (5′-TGTTGTTAGTTTTGGTTTAT-3′) were applied to detect the presence or absence of methylation.

## Results

### Re-expression of MLH1 and MAGE-A1 *in vitro* by decitabine and belinostat

**Figure 1:**
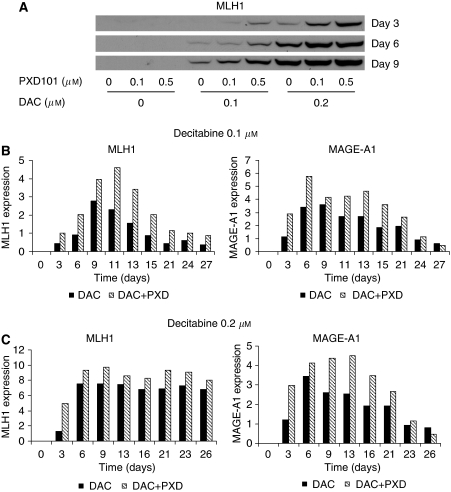
(**A**) MLH1 expression determined by western blotting of A2780/cp70 cell lysates prepared at various times after treatment of cells with DAC either alone or in combination with belinostat (PXD101). Cells were treated for 48 h (days 1 and 2) with decitabine with belinostat added for the second 24 h (day 2). MLH1 and MAGE-1a expression determined by western blotting and quantified by densitometry at various times after treatment of A2780/cp70 cells with either decitabine (**B**) 0.1 *μ*M, (**C**) 0.2 *μ*M; filled bars) or decitabine and belinostat (0.1 *μ*M; hatched bars). Results shown are representative of one of three experiments.

Treatment of MLH1 negative A2780/cp70 cells on days 1 and 2 with decitabine results in a dose-dependent re-expression of MLH1 as measured by western blot 3, 6 and 9 days after the start of treatment (Figure 1A). Belinostat treatment alone had no detectable effect on MLH1 levels. Treatment with decitabine on day 1 and with both decitabine and belinostat on day 2 results in a marked increase in MLH1 expression compared to treatment with decitabine alone on days 1 and 2. Re-expression of MLH1 was transient following treatment with decitabine at 0.1 *μ*M (Figure 1B) but was more sustained at 0.2 *μ*M (Figure 1C). Addition of belinostat increased the level of expression of MLH1 but did not alter the time course of re-expression or re-silencing. Decitabine treatment also induced re-expression of MAGE-A1 and again the expression was enhanced by the addition of belinostat. Re-expression of MAGE-A1 was transient at both concentrations of decitabine and belinostat and did not alter the time course of MAGE-A1 re-expression or re-silencing (Figure 1B and C).

### Re-expression of MLH1 and MAGE-A1 *in vivo* by decitabine and belinostat

**Figure 2:**
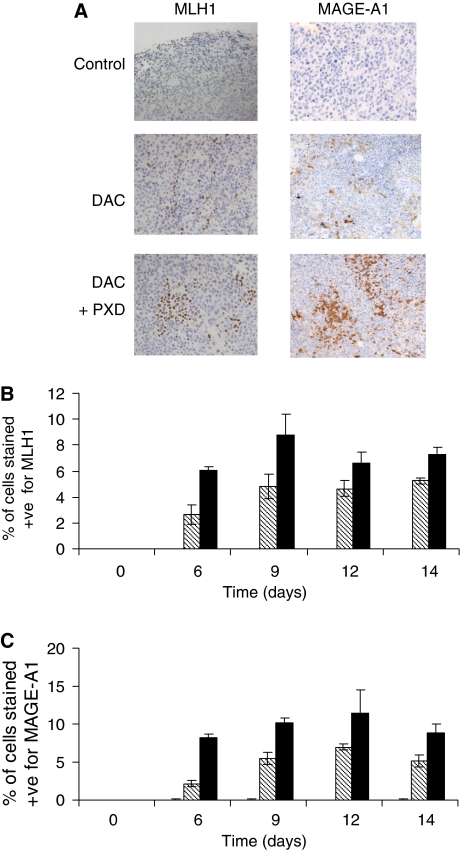
(**A**) MLH1 and MAGE-1A expression detected by immunohistochemistry in A2780/cp70 xenografts 12 days after treatment with decitabine (5 mg kg^−1^ × 3 on day 0) alone or followed by belinostat (40 mg kg^−1^ on day 3). (**B**) MLH1 and MAGE-1a expression at various times after treatment with belinostat (open bars), decitabine (hatched bars) or decitabine and belinostat (filled bars). (**C**) Expression is quantified as the percentage of cells that stain positive (+ve).

Treatment of mice with decitabine induces re-expression of MLH1 in A2780/cp70 xenografts and expression is maximal by about day 9 (Figure 2A and B). A similar time course is observed for MAGE-A1 expression (Figure 2C). Belinostat alone has no detectable effect on MLH1 and MAGE-A1 expression. The combination of decitabine and belinostat produces a marked increase in the level of re-expression of both MLH1 and MAGE-A1 to a greater extent than that achieved with decitabine alone (Figure 2). Gene re-expression is detectable by immunocytochemistry in only about 10% of cells and these cells appear in clusters following decitabine and belinostat treatment (Figure 2A, day 12).

### Methylation of MAGE-A1

**Figure 3:**
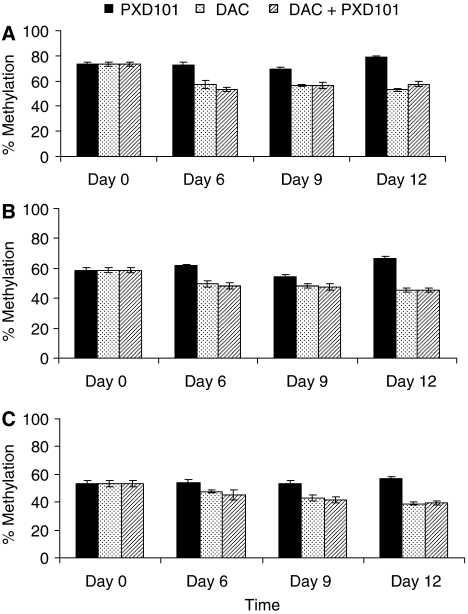
Pyrosequencing analysis of methylation of three CpG sites within a CpG island in the *MAGE-1a* gene promoter in A2780/cp70 xenografts. Mice were treated with decitabine alone on day 0 (5 mg kg^−1^ × 3; stippled bars), belinostat alone on day 3 (40 mg kg^−1^; filled bars) or with the combination of decitabine on day 0 and belinostat on day 3 (hatched bars). Tumours were removed and cytosine methylation measured on day 0 (untreated) and on days 6, 9 and 12. Cytosine methylation at three CpG sites within the MAGE-A1 promoter (CpG 17 (**A**), CpG 18 (**B**) and CpG 19 (**C**)) was determined.

Cytosine methylation was examined at 3 CpG sites within the MAGE-A1 gene promoter. At each site the level of methylation was reduced by decitabine treatment but there was no further reduction following treatment with decitabine and belinostat in combination (Figure 3). Although only between 6 and 20% demethylation is observed at these sites it should be noted that this will be an average throughout the cell population and only those cells, which are proliferating will incorporate decitabine and become demethylated. Global 5-methyl-2′-deoxycytidine levels in DNA from the tumours taken on day 6 was measured by HPLC (Appleton *et al*, 2007). Decitabine treatment reduced cytosine methylation (5-methyl-2′-deoxycytidine as a percentage of total 2′-deoxycytidine) from 3.43±0.16 in the control tumours to 2.78±0.05 (*n*=3, *P*<0.01) and this was not significantly different from the levels observed in the tumours taken from mice treated with decitabine in combination with belinostat (2.92±0.12, *n*=3) Belinostat treatment had no effect on 5-methyl-2′-deoxycytidine levels (3.70±0.28, *n*=3).

### Effects of decitabine and belinostat pre-treatment on drug sensitivity

**Figure 4:**
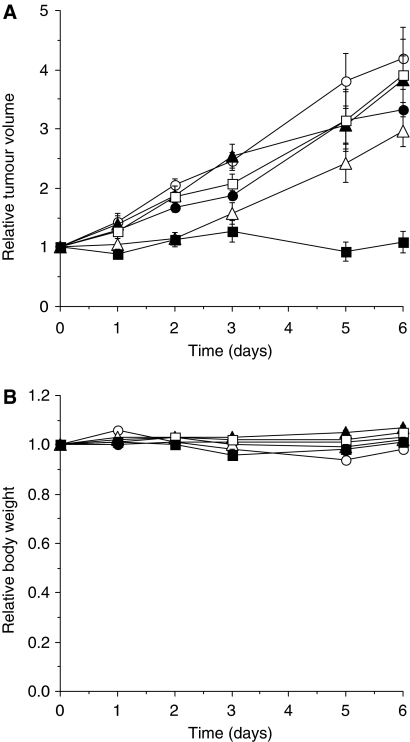
The effect of decitabine and belinostat pre-treatment on the drug sensitivity of A2780/cp70 xenografts. Mice were treated with decitabine (5 mg kg^−1^ × 3) 6 days before cisplatin treatment (day −6) and with belinostat (40 mg kg^−1^) 3 days before treatment with cisplatin (day −3). Cisplatin (6 mg kg^−1^) was administered on day 0. The groups are control (•; untreated); cisplatin alone (○; 6 mg kg^−1^ on day 0); decitabine alone (▴; 5 mg kg^−1^ × 3 on day −6); decitabine and cisplatin (▵; decitabine on day −6 and cisplatin on day 0); belinostat alone (□; 40 mg kg^−1^ on day −3); decitabine and belinostat followed by cisplatin (▪; decitabine on day −6, belinostat on day −3 and cisplatin on day 0). Results for (**A**) tumour volumes and (**B**) body weights are the means±s.e.m. of six mice.

A2780/cp70 is resistant to the maximum-tolerated dose of cisplatin *in vivo*. Treatment with decitabine or belinostat either alone or in combination has no effect on tumour growth and belinostat did not sensitise tumours to cisplatin. Pre-treatment of the mice with decitabine 6 days before treatment with cisplatin results in a significant growth delay and this effect is enhanced by the combination of decitabine and belinostat (Figure 4A). The treatments were well tolerated by the mice and there was no significant effect on body weight (Figure 4B).

## Discussion

We have shown clearly that the combination of low doses of decitabine and belinostat results in re-expression of epigenetically silenced genes and that when used *in vivo* in mice the combination can sensitise drug-resistant tumours to cisplatin more effectively than either drug alone.

We have already established that decitabine can be used to sensitise drug-resistant tumours to a number of clinically relevant cytotoxic drugs including cisplatin, carboplatin, epirubicin and temozolomide (Plumb *et al*, 2000). The inclusion of pharmacodynamic measurements in a phase 1 trial of decitabine and carboplatin has enabled us to show that decitabine can induce in patients the levels of demethylation in surrogate PBMCs seen in our mouse studies at doses that cause chemo-sensitisation (Appleton *et al*, 2007). However, it is also clear from the phase 1 trial that demethylation in PBMCs by decitabine is limited by the myelosuppressive activity of the drug. The level of demethylation observed in tumours was limited and heterogeneous between patients. In order to potentially increase the reversal of epigenetic silencing by decitabine, we have examined the addition of an HDAC inhibitor on re-expression of epigenetically silenced genes and chemo-sensitisation. Belinostat alone has no effect on MLH1 expression and this is consistent with the observation that histone deacetylase inhibitors cannot induce the expression of genes silenced due to promoter methylation (Suzuki *et al*, 2002). From the results with the cell line it is clear that belinostat can enhance the effects of decitabine on gene re-expression (Figure 1). Although belinostat increases the level of re-expression of both MLH1 and MAGE-A1 it does not appear to alter the kinetics of re-expression. This is consistent with the observation that the HDAC inhibitor 4-phenylbutyric acid does not inhibit re-silencing of p16 after decitabine treatment (Egger *et al*, 2007). Re-expression of MAGE-A1 is transient. It can be detected by 3 days after treatment but is lost after about 26 days. Re-expression of MLH1 is also transient at lower concentrations of decitabine (0.1 *μ*M), but is more sustained at the higher concentration (0.2 *μ*M) such that it remains detectable after 26 days. This may reflect the long half-life of the protein or may be due to a slower rate of gene re-methylation at the higher dose of decitabine.

To study the combination in human tumour xenografts we used the same schedule for decitabine that was shown to sensitise the tumours to cisplatin (Plumb *et al*, 2000) and attempted to improve on this response. Initial studies investigated the effects on gene re-expression and we have shown that a single dose of belinostat administered 3 days after decitabine treatment results in an increase in expression of both MLH1 and MAGE-A1 to a level greater that is seen with decitabine alone (Figure 2). MLH1 and MAGE-A1 gene re-expression is detected in about 6% of cells following treatment with decitabine and increases to about 10–12% when mice are treated with the combination of decitabine and belinostat. The apparent clustering of cells that re-express MLH1 and MAGE-A1 in the xenografts could represent areas of active proliferation within the tumours, which would be consistent with decitabine being incorporated into DNA during S-phase only and cell proliferation being required for demethylation. For the MAGE-A1 gene promoter decitabine treatment results in a decrease in the methylation of all three CpG sites studied (Figure 3). However, there is no further reduction in methylation following addition of belinostat to decitabine, which suggests that the enhanced gene expression observed with the combination is not due to direct effects on gene methylation. A study of the combination of decitabine and trichostatin A on MLH1 expression also concluded that the effect of the HDAC inhibitor was not due to a further reduction in DNA methylation (Cameron *et al*, 1999). It is possible that the HDAC inhibitor allows increased access of the transcription factors to the demethylated gene as a result of increased levels of histone acetylation and the resulting chromatin remodelling (Egger *et al*, 2007).

Re-expression of MLH1 is clearly apparent by 6 days after treatment and is maximal by about 9 days. As A2780/cp70 is a rapidly growing tumour, we treated with the cytotoxic drug as early as possible after decitabine treatment (day 6). This is the schedule used previously for decitabine alone (Plumb *et al*, 2000). A2780/cp70 xenografts are resistant to cisplatin. However, treatment with decitabine sensitises the tumours to cisplatin and the growth delay is further enhanced by the addition of belinostat (Figure 4). These results give clear support to the proposal to use decitabine in combination with a histone deacetylase inhibitor to enhance the chemo-sensitisation observed with decitabine alone.

Neither decitabine, belinostat nor the combination had any effect on tumour growth. This is not surprising as we have not attempted to use these drugs in the optimal schedule for antitumour activity. We have already shown that A2780/cp70 is sensitive to belinostat when mice are treated daily for 7 days (Plumb *et al*, 2003). As the aim was to combine the epigenetic therapies with a cytotoxic drug we have intentionally used low, non-toxic doses of the agents. Although decitabine treatment results in a reduced MAGE-A1 methylation in PBMCs the gene is not re-expressed and this may be due to a lack of the necessary transcriptional activators (Karpf *et al*, 2004). Few studies have examined the effects of demethylating agents on normal cells; however, there is some evidence that fewer genes become demethylated than in tumour cells (Liang *et al*, 2002). This suggests that epigenetic therapies will not necessarily be associated with genome-wide effects in normal tissues. Furthermore, the combination of a low dose of decitabine and the HDAC inhibitor phenylbutyrate has been shown to inhibit carcinogen-induced lung tumours in mice (Belinsky *et al*, 2004). This raises the possibility that in addition to sensitising drug-resistant tumours to chemotherapy the epigenetic therapy might also protect the normal tissues from some of the damage caused by the cytotoxic agent.

As the dose of decitabine that can be given to patients and hence the maximum pharmacodynamic effect as a demethylating agent is limited by toxicity and eventual re-methylation of genes, we suggest that the combination of decitabine and belinostat could have a role in increasing the efficacy of chemotherapy in tumours that have acquired drug resistance due to DNA methylation and gene silencing.
